# Insights into the molecular profiles of adult and paediatric acute myeloid leukaemia

**DOI:** 10.1002/1878-0261.12899

**Published:** 2021-02-11

**Authors:** Myint Myat Khine Aung, Megan L. Mills, Joana Bittencourt‐Silvestre, Karen Keeshan

**Affiliations:** ^1^ Paul O’Gorman Leukaemia Research Centre Institute of Cancer Sciences University of Glasgow UK

**Keywords:** acute myeloid leukaemia, adult, chemoresistance, clonality, omic profiling, paediatric

## Abstract

Acute myeloid leukaemia (AML) is a clinically and molecularly heterogeneous disease characterised by uncontrolled proliferation, block in differentiation and acquired self‐renewal of hematopoietic stem and myeloid progenitor cells. This results in the clonal expansion of myeloid blasts within the bone marrow and peripheral blood. The incidence of AML increases with age, and in childhood, AML accounts for 20% of all leukaemias. Whilst there are many clinical and biological similarities between paediatric and adult AML with continuum across the age range, many characteristics of AML are associated with age of disease onset. These include chromosomal aberrations, gene mutations and differentiation lineage. Following chemotherapy, AML cells that survive and result in disease relapse exist in an altered chemoresistant state. Molecular profiling currently represents a powerful avenue of experimentation to study AML cells from adults and children pre‐ and postchemotherapy as a means of identifying prognostic biomarkers and targetable molecular vulnerabilities that may be age‐specific. This review highlights recent advances in our knowledge of the molecular profiles with a focus on transcriptomes and metabolomes, leukaemia stem cells and chemoresistant cells in adult and paediatric AML and focus on areas that hold promise for future therapies.

## Graphical abstract

Acute myeloid leukaemia (AML) is a clinically and molecularly heterogeneous disease characterised by uncontrolled proliferation, perturbed myeloid differentiation and acquired self‐renewal of hematopoietic stem and myeloid progenitor cells. This review highlights recent advances in our knowledge of the molecular profiles of adult and paediatric AML with a focus on transcriptomes and metabolomes, leukaemia stem cells and chemoresistant cells.

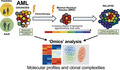

**Abbreviations**
AML: Acute myeloid leukaemia
APL: acute promyelocytic leukaemia
ATO: arsenic trioxide
ATRA: all‐trans‐retinoic acid
CH: clonal haematopoiesis
ES: enrichment score
FAO: fatty acid oxidation
GSEA: Gene Set Enrichment Analysis
HSCs: haematopoietic stem cells
ITD: internal‐tandem duplications
LSC: leukaemia stem cell
MDS: myelodysplastic syndromes
MPN: myeloproliferative neoplasms
MRD: minimal residual disease
NGS: next‐generation sequencing
pLSC6: paediatric 6 gene LSC score
TARGET: Therapeutically Applicable Research to Generate Effective Treatments
VAF: variant allele frequencies

## 1 Introduction

Acute myeloid leukaemia (AML) is a haematological malignancy, with incidence rates having grown by 29% since the early 1990s (www.cancerresearchuk.org). Incidence increases with age, and 70% of patients diagnosed with the disease still die from this disorder. The current overall survival rate in children is only 60–70% and thereafter falls progressively with age to < 5% in those aged over 65 years. Both children and adults die within 5 years from diagnosis due to a combination of relapse (up to 35% and 99%, respectively [1], and treatment‐related mortality [2].

To date, most studies on AML pathogenesis have focused on adult patients. AML is characterised by clonal expansion of myeloid blasts cells with uncontrolled proliferation [3]. It arises from leukaemic stem cells (LSCs) that maintain the disease [4] and that give rise to the clones of AML blasts. AML either develops *de novo* or appears as secondary disease, subsequent to underlying myeloproliferative neoplasms (MPN) or myelodysplastic syndromes (MDS). Mutations acquired over time can lead to clonal enrichment in the haemopoietic compartment in the absence of any clinical manifestations termed clonal haematopoiesis (CH). Such clones may then acquire subsequent mutations transforming into malignant clones that underlie the development of AML. In children, the vast majority of patients present with *de novo* AML, whilst in adults, AML more commonly arises from an underlying MPN or MDS, and this characteristic increases with age [5, 6]. Further age‐related sequential acquisition of mutations during the course of disease could largely explain the clonal heterogeneity in majority of adult AML with underlying MPN or MDS. Indeed, accumulation of somatic mutations in haematopoietic stem cells (HSCs) is proportional with age suggesting that the initial mutation for AML occurs stochastically in a cell which goes on to garner a unique combination of other AML‐related mutations by chance as an individual grows older [7]. In adults, it is possible there already exists haematopoietic cells with leukaemia somatic variants that are thriving as passenger lesions [8]. These primitive mutations could influence the type and order of further mutations and the evolution of AML clonal heterogeneity [9]. On the other hand, the features associated with ageing cannot be the explanation for paediatric AML incidence and clonal diversity. Thus, it seems the pathogenesis of AML in children may differ to that in adults.

Biology‐based ‘omics’ technologies have revolutionised the field’s comprehension of AML pathogenesis. The first AML genome was sequenced by Tim Ley *et al*. [10]. Since then, advancements in technologies have enabled genomic profiling (Box 1) down to the single‐cell level and high‐throughput analysis of large numbers of samples at an affordable cost. Distinct genetic and epigenetic alterations constitute the clonal diversity each individual case of AML presents [11]. The genomic information affects the unique morphology and immunophenotypic attributes of each clonal cell in a single AML case [12]. During disease advancement, the prevalence of malignant clones and subclones may change due to evolutionary pressures [13]. Genomic profiling is highly valuable for AML treatment strategies (Box 2), and additionally, some mutations and chromosomal aberrations are excellent for minimal residual disease (MRD) monitoring. Transcriptomics using bulk or single‐cell RNA sequencing followed by pathway analysis is a powerful analytical approach that can provide important insights in AML biological and biochemical features. This has been especially powerful in defining stem cell populations, chemoresistance and metabolic features of AML cells, which will be discussed further below.

Box 1 **Genomic Profiling**

Genomic profiling is a technique that enables us to explore the genetic information of an individual or in a particular cell type and the interactions in which these genes have with each other and the environment. The advent of genomic methodologies from microarrays to large scale genome sequencing has facilitated the study of not only genes but even the subsequential transcripts and proteins simultaneously [13]. In recent years, next‐generation sequencing (NGS) approaches such as whole‐genome sequencing and whole exome sequencing have largely replaced the preceding techniques (reviewed in Ref. [14]. Single‐cell DNA sequencing (scDNA‐seq) is a new and promising approach for high‐throughput study of clonality in AML with ever advancing technology such as the use of droplet microfluidics to analyse a few thousands of AML single cells [15]. Despite being compelled to technical artefacts such as high allelic dropout rates and random transcript coverage, scDNA‐seq is able to resolve the complicated subclonal structure of AML that cannot be defined through bulk analysis using variant allele frequencies (VAF) or inconclusive zygosity states.

Box 2 **Brief overview of AML treatment**

A small number of therapies targeted to mutational events and/or genomic profiles have been recently developed for patients with AML, although the standard of care treatment dates back to the 1970s [16]. The current standard induction chemotherapy protocols used in AML, often termed ‘3 + 7 regimens’, consist of an anthracycline (daunorubicin or mitoxantrone) and cytarabine (Ara‐C). New therapies for AML are extremely limited and are often only suitable for specific subgroups of patients, based on synthetic lethality in drug treatments or genetic features. This includes all‐trans‐retinoic acid (ATRA) targeted therapy for *RAR* rearranged AML, FLT3 inhibitors for *FLT3* mutated subtypes, IDH1/2 inhibitors for *IDH* mutated groups and hypomethylating agents. For example, Enasidenib and ivosidenib have been approved by the Food and Drug Administration in 2017 and 2018 for the treatment of adult relapsed/refractory AML with IDH2 and IDH1 mutations, respectively. ATRA plus arsenic trioxide (ATO) has recently been approved as frontline therapy for acute promyelocytic leukaemia (APL) [17]. APL is a type of AML characterised by the PML‐RARα fusion oncoprotein. ATRA and ATO treatment induces the degradation of PML‐RARα oncoprotein. Indeed, two epigenetic compounds azacitidine and decitabine, inhibitors of DNA methyltransferases, have been recently approved for use clinically in myeloid cancers including AML [18, 19]. Many other epigenome targeted compounds are in early stages of clinical trials for translational application. Moreover, given the inherent reversibility of epigenomic marks [20], identifying biomarkers, with the help of the molecular profiling tools, that can be exploited for patient prognostics to particular targeted therapies will be instrumental for making informed decisions in treating AML of distinct subtypes. More recently, BCL‐2 inhibitor venetoclax has been approved for clinical use [21]. It is unclear whether the novel therapies recently approved for adult AML will be suitable for paediatric cases, at all, or only in a minority of cases given the distinct genetics, frequencies of mutations and transcriptional profiles of paediatric AML [22, 23]. Additionally, what is emerging now in adult AML are features of chemoresistant cells, notably in their transcriptional profiles, that distinguish them from therapy‐naïve cells [26, 27] and which may be more suitable for therapeutic targeting rather than the mutation *per se*. Chemoresistant profiles of AML should be considered in terms of the age the patient and the associated genetics, given the above mentioned differences in paediatric and adult AML. It is clear that some mutations and chromosomal aberrations are excellent for minimal residual disease (MRD) monitoring [28, 29]. However, it is also clear that those markers persist following treatment as in the case of RUNX1‐RUNX1T1 in the absence of clinical manifestation of the disease. Designing novel therapies that target the chemoresistant transcriptional profile that could be combined with the mutation/fusion protein targeting drug or used as an alternative to standard chemotherapy is worth considering. In a recent study of primary chemotherapy resistance in a cohort of 107 children and adults with AML using targeted gene sequencing, it was shown that few patients exhibited specific individual mutations associated with primary chemotherapy resistance and failure of induction chemotherapy [30]. Therefore, it appears that additional genetic or molecular mechanisms mediate chemotherapy resistance in paediatric and adult AML.

In this article, we review genetic, epigenetic, transcriptional and metabolic profiles and their association with clonal heterogeneity, whilst highlighting differences between paediatric and adult AML. Next, we focus specifically on molecular AML profiles and chemoresistance, as the cells that survive chemotherapy have recently been shown to be transcriptionally distinct from therapy‐naïve cells. We will also consider the molecular profiles that may be used for developing new therapies in the context of specific populations such as LSCs and chemoresistant cells.

## 2 Genetic and epigenetic profiles of paediatric and adult AML

**Fig. 1:**
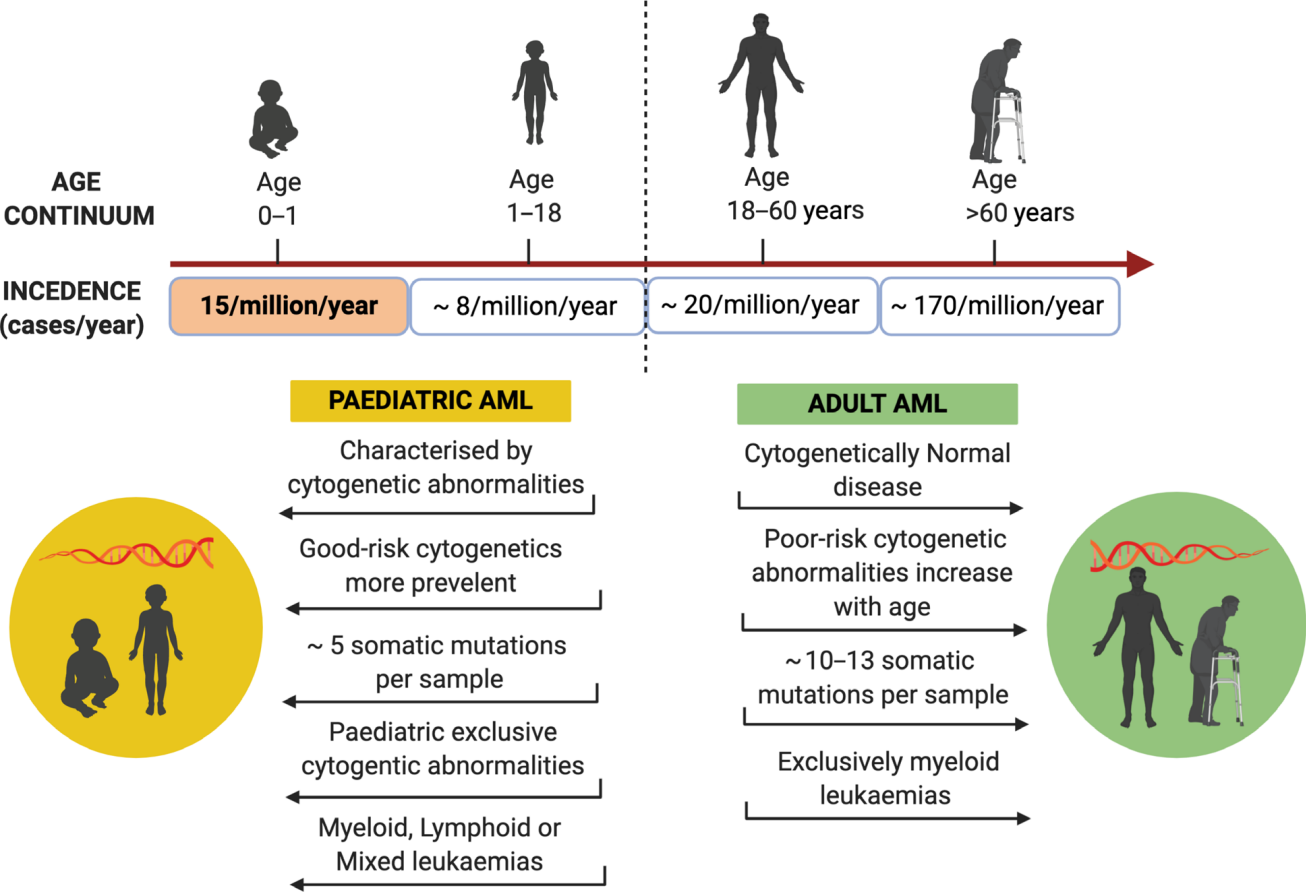
Schematic illustration of the skewed pattern of AML incidence rates (cases/year) and the distinct cytogenetic and molecular features that exist across the age‐spectrum.

The genetic landscape and mutational load (Box 3) are very different between children and adults (Fig. 1). Only 20% of paediatric patients have a normal karyotype, with the majority bearing chromosomal abnormalities, and the number of somatic mutations in paediatric patients is lower than in adult patients (5–6 somatic mutations per paediatric sample [5, 31, 32] vs 10–13 per adult genome [33, 34]. Cytogenetic and sequencing studies of paediatric and adult AML have revealed significant age‐specific genomic differences [2, 31, 34, 35, 36, 37] (amongst others which have been reviewed in detail elsewhere). Adult AML has been classified with at least 11 genetic classes [34], and over 20 subsets can be assigned when also considering cell differentiation states of the leukaemic blasts [2, 38]. Similar trends in mutated gene co‐occurrence and mutational hotspots are reported in the paediatric population [31]; however, the extent to which the site, frequency and co‐occurrence of specific mutations identified are distinct from those in adult AML. In brief, in paediatric patients, mutations in *FLT3, NPM1, WT1, CEBPA* and *KIT* are most frequent, *RUNX1, CBFB* and *KMT2A* (a.k.a MLL) fusions most commonly occur as well as structural aberrations including trisomy 8 and loss of the Y chromosome. The incidence of KMT2A‐rearranged AML is much higher in children compared to adults (38% vs 2%, respectively), with the highest incidence in infants (77%) [1, 6]. Alterations in *RAS*, *KIT* and *WT1* genes are more common in children than adults. In contrast, mutations in *DNMT3A* and *TP53,* which are commonly found in adults, are virtually absent in paediatric patients. More common mutations in adult AML are *NPM1* and genomic subgroups consisting of AMLs with mutated chromatin and RNA‐splicing genes (e.g. *SRSF2, DNMT3A, TET2*) and class defining *IDH2* mutations. The frequency of adult patients with *RUNX1, CBFB* and *KMT2A* fusions is low, whereas complex karyotypes are frequent. *FLT3* mutations commonly occur across the age groups, with similar frequency. However, unique and paediatric‐specific *FLT3* variants have been reported [39].

Box 3 **Mutational Profiling**

It is common that AML patients exhibiting similar clinical presentations have substantially different mutational aberrations with varying clinical outcomes to therapies [40]. In 2017, the European LeukaemiaNet [2] factored in the significance of mutational events in AML and improved the predictive strategy for response to therapy and survival by adding recently recognised mutations in *RUNX1*, *ASXL1* and *BCR‐ABL1* to the known risk contribution delimited by mutations in *NPM1*, *FLT‐ITD*, *CEBPA* and *TP53*. Within this risk stratification system, patients are classified according to the cytogenetic and molecular characteristics into three prognostic classes – adverse risk, intermediate risk and favourable risk. However, there could be many more genes that could be substantial for leukaemia development as well as apprise the appropriate therapies for individuals, and the fast advancing molecular profiling assays hold the power to delve into and discover these genes.

Changes in the epigenetic landscape are a feature of adult AML, and various regulators for the AML epigenome are subjected to mutation, deletion and chromosomal translocation [41]. Examples of epigenetic variations include cytosine methylation patterning, histone proteins alterations and RNA‐associated gene silencing [20]. Multiple epigenetic profiling efforts have observed recurrent mutations in genes that govern these processes such as DNMT3A, TET2, IDH1/2, although they arise fewer in children than in adults and are usually early events in leukaemogenesis [22]. Associated with specific genetic subgroups are distinct transcriptional states [42], and in this report, we will highlight transcriptional profiling in paediatric and adult AML.

## 3 Transcriptional profiles of paediatric and adult AML

**Fig. 2:**
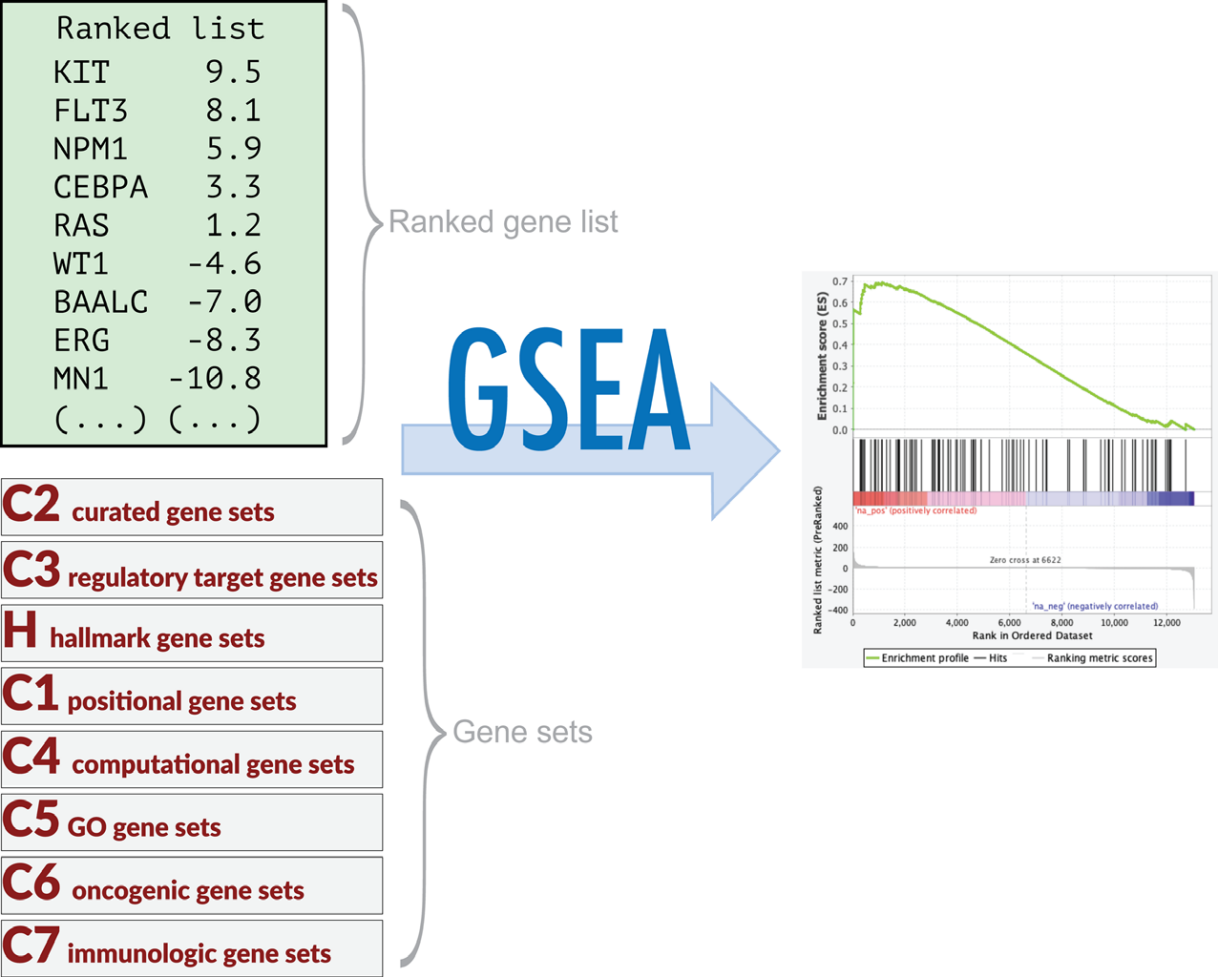
Exemplar for pathway analysis workflow. After differential expression analysis the list of differentially expressed genes can be used for GSEA as a preranked gene list (other input options are also available). Depending on your research interests, one or more gene sets can be selected and GSEA will calculate a score reflecting whether each gene set is overrepresented at the extremities of the ranked gene list. The graph shows the enrichment profile, and the enrichment score (ES) is the point furthest from zero. ES values, normalised ES, false discovery rate and other information are contained in the results report.

There is a growing list of data sets of transcriptional AML profiles, and we have highlighted some that are publically available that we have mentioned in this review (Table 1 contains a nonexhaustive list, Fig. 2). One of the limiting factors in assessing the distinct molecular features of paediatric and adult AML is the availability of data sets that contain AML specimens across the age continuum or of data sets that contain comparable AML subgroups from adults and children. Comparative analysis across different platforms (e.g. microarray and RNA‐seq, see Table 1 containing a representative list for available data sets) is possible but does come with additional computational limitations. Recently, a large data set that enables comparative analysis of AML across the ages has been made available: Tyner *et al*. published on the BEAT AML cohort (Box 4) consisting of 572 AML patients across the age continuum (18 patients < 19 years, 54 patients 20–35 years, 136 patients 36–55 years, 141 patients 56–65 years and 207 patients > 66 years) [33].

**Table 1:** Transcriptomic data sets within references cited in this review and data sets used in our lab (nonexhaustive list of those publically available). For AML type, French classification nomenclature was preferentially used and, when not available, cytogenetics was used instead. AMKL, acute megakaryoblastic leukaemia; CN‐AML, cytogenetically normal AML; LSK, Lin^−^SCA1^+^c‐KIT^+^; PB, peripheral blood.

Data set ID	Location	Species	Age	Cell type	AML type	Treatment	Type of data	Reference
AML1	https://www.stjuderesearch.org/site/data/AML1/	Human	Paediatric and Adult	Primary BM, PB	M0‐M7	None	Microarray	Ross *et al*. (2004) [119]
GSE1159	GEO	Human	Adult	Primary BM, PB	AML (M0 – M7) and healthy	None	Microarray	Valk *et al*. (2004) [42]
GSE4119	GEO	Human	Paediatric and Adult	Primary BM, PB	AMKL	None	Microarray	Bourquin *et al*. (2006) [120]
GSE5258	GEO	Human	Adult	Cell lines MCF7, HL60, ssMCF7, PC3, SKMEL5	AML M2, Breast adenocarcinoma, prostate carcinoma, melanoma	164 small molecules	Microarray	Lamb *et al*. (2006) [104]
GSE17061	GEO	Human	Unknown	Primary BM, PB	AML‐M0	None	Microarray	Silva *et al*. (2009) [121]
GSE6891	GEO	Human	Adult	Primary BM, PB	M0‐M6 and nondetermined	None	Microarray	Verhaak *et al*. (2009) [122]
GSE24006	GEO	Human	Adult	Primary BM, PB, cord blood	AML and healthy	None	Microarray	Gentles *et al*. (2010) [123]
GSE17855	GEO	Human	Paediatric	Primary BM, PB	MLL‐rearrangements, t(8;21)(q22;q22), Inv(16)(p13;q22), t(15;17)(q21;q22), t(7;12)(q36;p13), CN‐AML, etc.	Patients treated with Cytarabine and anthracycline	Microarray	Balgobind *et al*. (2011) [124]
GSE30377	GEO	Human	Unknown	Xenotransplanted Primary BM cells	Unknown	None	Microarray	Eppert *et al*. (2011) [84]
GSE64776	GEO	Human	Adult	Primary cells	MLL	None	Microarray	Iwasaki *et al*. (2015) [90]
GSE64773	GEO	Mouse	Unknown	Primary cells	HoxA9 Meis1 shRNA induced AML and healthy	None	Microarray	Iwasaki *et al*. (2015) [90]
GSE74666	GEO	Human	Paediatric	Cell line MV4‐11	AML M5 t(4;11)(q21;q23)	Sorafenib	Microarray	Ref. [125]
GSE76009	GEO	Human	Adult	Primary BM, PB, peritoneal fluid	APL, non‐APL	None	Microarray	Ng *et al*. (2016) [85]
GSE81842	GEO	Mouse	Unknown	Primary BM, PB, spleen, gonadal adipose tissue	BCR‐ABL and NUP98‐HOXA9	None	RNA‐Seq	Ye *et al*. (2016) [116]
GSE92778	GEO	Human	Adult	Cultured Primary BM cells from patients	AML and healthy	None	Microarray	Boyd *et al*. (2017) [126]
GSE107662	GEO	Mouse	0 – 52w	Primary FL, BM	NUP98‐HOXA9 retrovirus	None	RNA‐Seq	Chaudhury *et al*. (2018) [23]
GSE122066	GEO	Mouse	3w, 52w	Primary BM	LSK, non‐AML	None	RNA‐Seq	Chaudhury *et al*. (2018) [23]
GSE114111	GEO	Human	Adult	Cell line MOLM‐13	ins(11;9)(q23;p22p23)	FIS1‐shRNA	RNA‐Seq	Pei *et al*. (2018) [80]
GSE114109	GEO	Human	Adult	Primary BM, PB	Multiple cytogenetics	FIS1‐shRNA	RNA‐Seq	Pei *et al*. (2018) [80]
GSE114105	GEO	Human	Adult	Primary BM, PB	Multiple cytogenetics	PRKAA1‐shRNA	RNA‐Seq	Pei *et al*. (2018) [80]
GSE116567	GEO	Human	Adult	Primary BM, PB	Multiple cytogenetics	Untreated, venetoclax and azacitidine	RNA‐Seq	Jones *et al*. (2018) [79]
GSE134506	GEO	Human	Adult	Cell line HEL	AML M6	Doxorubicin, Cytarabine, Decitabine	Microarray	Caiado *et al*. (2019) [71]
TARGET	https://ocg.cancer.gov/programs/target/data‐matrix	Human	Paediatric	Primary cells	t(6;9), t(8;21), t(3;5)(q25;q34), t(6;11)(q27;q23), t(9;11)(p22;q23), t(10;11)(p11.2;q23), t(11:19)(q23:p13.1), inv(16), del5q, del7q, del9q, MLL	None	RNA‐Seq and microarray	Duployez *et al*. (2019) [92]
E‐MTAB‐7729	Array Express	Human	Paediatric	Primary cells	TAM and ML‐DS	None	Microarray	Labuhn *et al*. (2019) [45]
GSE116256	GEO	Human	Adult	Primary BM, cell lines MUTZ‐3, OCI‐AML‐3	AML and healthy	Untreated and treated patients (multiple drugs)	scRNA‐Seq	van Galen *et al*. (2019) [67]

Box 4 **BEAT AML**

The Beat AML program involved 11 academic medical centres who worked collectively to accrue a cohort of ~ 950 AML patient specimens and 11 pharmaceutical and biotechnology companies, who supplied drugs for testing. These specimens were subjected to whole exome sequencing, RNA sequencing and ex vivo drug sensitivity analyses. The majority of this data set was published in October 2018 in Nature [33]. The authors provided detailed clinical annotations, genomic and transcriptomic analyses and ex vivo drug sensitivity studies, and analytical approaches for data integration and accessible through the Beat AML data viewer http://www.vizome.org/).

The use of mouse models to address the molecular differences between paediatric and adult AML is equally challenging, given the myriad of biological variables that should be considered in the experimental set‐up, even within a defined genetic subgroup, including age‐specifications for cell of origin, cell hierarchy, cell surface marker expression, cell cycle, microenvironment and localisation. Nevertheless, mouse models featuring specific genetic AML features (i.e. NUP98‐HOXA9, ETO2‐GLIS2) have shown that paediatric and adult AML are distinct biological entities, depending on the age of the cell of origin and whether it is of fetal or adult origin [23, 43]. The data suggest that the transcriptional programming within the cell of origin (fetal or adult) can explain the differences in paediatric and adult disease development. Indeed, it has been speculated that the *in utero* mutational acquisition (such as GATA1s mutation in trisomy 21‐AML) may have to do with the transcriptional state in the fetal liver, as this mutation does not occur later in life [44, 45].

## 4 Metabolic profiles of paediatric and adult AML

Distinct metabolic alterations have been increasingly reported in adult AML following the advent of metabolomic profiling (Box 5). For example, a study conducted by Chen *et al*. identified an altered glucose metabolism signature in adult patients with AML, in which six associated glucose metabolism markers were described to significantly inform upon patient prognosis. However, this phenomenon has not yet been reported among paediatric cohorts [46]. Of note, *IDH1/2* mutated adult AML has been described to drive aberrant perturbations in the tricarboxylic cycle through the reduction of alpha‐ketoglutarate to the oncometabolite 2‐hydroxyglutarate [47]. Comparatively, AML internal‐tandem duplications (ITD) in the *FLT3* gene confers dependence on aerobic glycolysis via a marked upregulation of the mitochondrial gate keeper enzyme, hexokinase‐2 [48]. To date, few studies have attempted to investigate the metabolome of paediatric AML. As a result, there is currently a gap in our knowledge of metabolic vulnerabilities significant to paediatric AML that could guide future lines of preclinical investigation for targeted treatment. Furthermore, the uncontrolled growth of leukaemic cells and the development of chemoresistance is now increasingly affiliated with the systemic supply of nutrients for amino acid and fatty acid metabolism [49, 50]. Homing in on the stark alterations in metabolism between leukaemic cells and ‘normal’ haematopoietic cells currently represents a promising avenue in patient diagnosis and the development of more targeted and personalised therapies [51, 52, 53]. Research into novel AML therapies has highlighted the contributions of cellular metabolic adaptations in promoting both resistance to conventional chemotherapy and targeted inhibition in leukaemic cells [54].

Box 5 **Metabolic profiling**

Metabolic reprogramming is a common cancer cell phenotype in which neoplastic cells acquire vast amounts of energy and metabolites to sustain uncontrolled growth and proliferation. Driven by an array of extrinsic bone marrow (BM) microenvironmental cues and intrinsic malignant cell factors, metabolic reprogramming has given rise to a range of context‐specific metabolic phenotypes [55]. Metabolomic profiling is emerging as a systemic technology that focuses on the comprehensive and semiquantitative analysis of metabolites and small molecule intermediates within specific cells, tissues and other biological specimens [56]. Metabolomics platforms, including proton nuclear magnetic resonance spectroscopy and mass spectrometry, have provided a robust and innovative approach to study the AML metabolome through the characterisation of by‐products that reflect biologically relevant changes in cellular state.

## 5 Clonal heterogeneity and chemoresistance in adult and paediatric AML

Although AML can be preceded by CH, most of CH clones are not ‘preleukaemic’ and will never transform to AML. Distinguishing benign CH from preleukaemic CH is a clinical goal in order to prevent AML transformation. Recently, p53 conformational changes in *WT p53 DNMT3A* mutant clones were shown to distinguish benign CH from true preleukaemia (EHA abstract S133, 2020, Tuval and Schlush). The number, identity and burden of mutations in CH are associated with the risk and timing of progression to AML [57, 58]. Common CH‐associated mutations include *DNMT3A, TET2* and *ASXL1* (‘DTA’ mutations) and, despite being found in AML, are not helpful for MRD monitoring as these DTA mutations are not lost even in remission [28]. Somatic aberrations that disrupt the cytosine methylation system (epigenetic) such as mutations in *DNMT3A* and *TET2* occur in preleukaemic cells early and may give proliferative favour, but are inadequate in causing the disease [59]. AML initiating mutations in the *NPM1* gene, however, do manifest in AML [60] and are neither preleukaemic nor CH‐related. Thus, MRD monitoring of NPM1‐mutant clones may be combined with analysis of prevailing preleukaemic mutations in clones at remission to objectively enhance or even replace therapy‐naïve genomics to predict clinical outcomes [29, 61]. ‘Preleukaemic’ clones arise with surprising frequency during fetal development also. The *in utero* acquisition of leukaemogenic fusions involving the *KMT2A* and *RUNX1* genes [62, 63] is frequently seen, at a much higher rate, however, than the development of childhood AML with these fusions. This indicates clonal expansion of cell populations with these leukaemic fusions, which are not sufficient for disease development. This has been supported by transgenic studies, for examples those showing a lack of disease with *RUNX1* fusions without additional mutations [64]. Indeed in the case of RUNX1‐RUNX1T1 fusions, it is known such clones can persist for years in the absence of disease, such as in patients that have received treatment for RUNX1‐RUNX1T1‐positive AML and remained in remission despite the persistent detection of RUNX1‐RUNX1T1‐positive cells [65, 66].

The subclonal architecture of AML at diagnosis and relapse has been modelled by several groups in an attempt to understand both the evolution of the disease and the factors contributing to treatment resistance [67, 68, 69] (Fig. 4A). Using NGS on AML patients of *FLT3‐ITD*‐specific subgroup at three different time points, diagnosis, complete remission and relapse revealed that clonal evolution occurred in either two ways – (a) a dominant clone that acquired new somatic aberrations due to DNA damaging therapeutic agents and expanded in relapse or (b) subclones pre‐existing in low numbers at diagnosis evaded killing and prevailed as the dominant clone at relapse [70]. Lineage tracing of DNA barcodes of adult AML cohort combining exomic, transcriptomic and phenotypic profiling revealed a group of preordained barcoded clones with common molecular ancestry constituting gene expression or functionally construed stemness and chemoresistant properties that are positively selected upon chemotherapy exposure [71].

A greater number of paediatric patients present with *de novo* AML than in adults and rarely have the common adult mutations such as *DNMT3A* and *TET2* [5] suggesting that a difference in clonal heterogeneity between adult and paediatric cases may be explained by the genetic mutations. The TARGET initiative study applied whole exome sequencing on 20 *de novo* paediatric AML cases following matched trios of samples at diagnosis, complete remission and relapse and highlighted compelling clonal evolution and diversity with persistence (diagnostic VAF > 0.4), loss (diagnostic VAF < 0.2) and gain of molecular events using sequence and copy number alterations data, as shown in adult AML data [32]. McNeer *et al*. [72] (another TARGET initiative study on paediatric AML) employed whole‐genome and RNA sequencing found that AML groups with genetic clones bearing initiation mutations such as *NUP98‐NSD1* collude with mutations in *FRMD8, WT1* and others to prevail and may correlate with causing chemoresistance, whereas subclones with mutations in *FLT3* and *NRAS* were eradicated by chemotherapy and thus are not associated with disease relapse after chemotherapy. Since there is a lack of research on clonality and chemoresistance in paediatric AML, it is exciting to anticipate what future studies that specifically quantify age‐related clonal diversity may bring.

Intratumoral heterogeneity has been extensively studied using cell surface markers [73]. However, this approach relies on predefined markers that may not accurately represent underlying transcriptional programmes nor accurately represent paediatric AML heterogeneity. Recently advances in single‐cell RNA‐sequencing (scRNA‐seq) technologies have enabled the analysis of single‐cell transcriptional states together with the genetic background [67]. In this paper, the authors present methods for amplifying barcoded transcripts of genes that are frequently mutated in AML. AML cells could be distinguished into AML cell types and differentiation states and importantly could be separated from normal cells. Over the clinical course however following treatment, their identity as normal or malignant required adaptation of the methodology and it had to be assumed that the mutation was present postchemotherapy as well as at diagnosis. It could be argued that clones/single cells that emerge postchemotherapy may not have the diagnostic mutation and are therefore missed by this assumption. For example, *FLT3* mutations are unstable and can be gained or lost as the disease progresses, with patients exhibiting *FLT3* status shift from diagnosis to relapse in 1–30% cases [2, 74, 75]. Indeed, mutations of *FLT3* exhibited relative depletion by chemotherapy in AML induction failure suggesting that its subclonal evolution in and of itself does not cause chemotherapy resistance. Rather, its activation in combination with specific other pathogenic events as part of distinct clones such as those with mutations of *WT1* or *NUP98* rearrangements or others may cause resistance to chemotherapy.

Significant advances have been made in identifying geneticially and transcriptionally distinct AML subclones using an unbiased proteomic analysis of CD34^+^ AML‐specific plasma membrane proteins [76]. Using this approach, clones that could be distinguished based on surface membrane expression of CD25 or IL1RAP could be separated into distinctly evolved clones that had acquired different additional mutations and different transcriptional profiles, notably in metabolic programmes such as ROS, OXPHOS and glycolytic pathways.

## 6 Adult and paediatric AML leukaemic stem cells and chemoresistant cells

### 6.1 LSC profiles and prognostic value in adult and paediatric AML

**Fig. 3:**
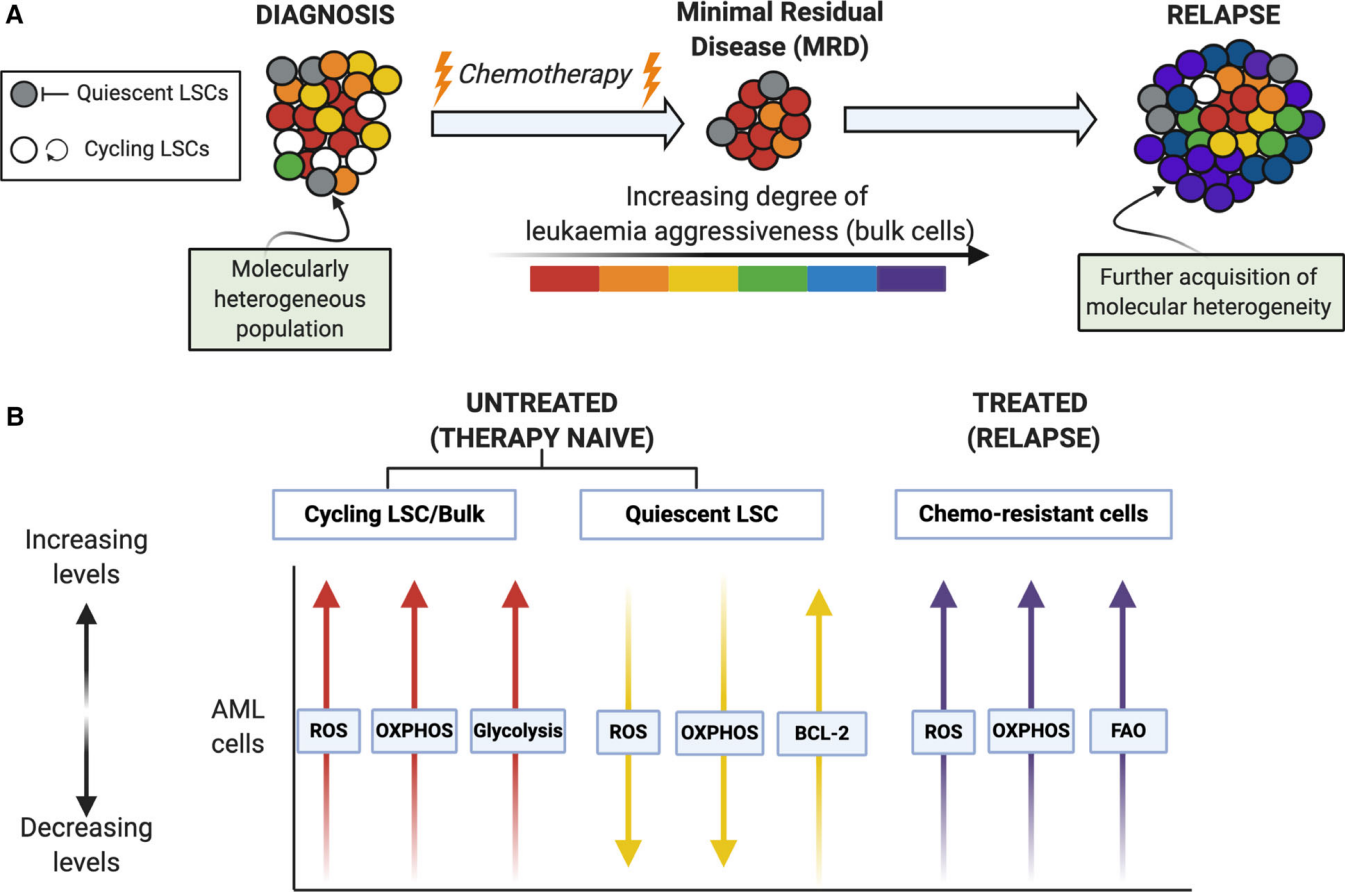
Schematic representation of cellular and metabolic changes in adult AML following chemotherapy. (A) At diagnosis, AML comprises a molecularly heterogeneous population of cycling and quiescent LSCs and bulk AML blast cells. Treatment with conventional chemotherapy often results in complete remission; however, there many remain small numbers of leukaemic cells that are chemoresistant giving rise to what is termed ‘minimal residual disease’ (MRD). The MRD chemotherapy‐resistant LSC population possess the ability to re‐initiate leukaemia and potentiate disease aggressiveness through further acquisition of molecular heterogeneity. (B) The associated metabolic programmes of LSCs and bulk cells are dynamic and vary based upon therapy status. Initially, both therapy‐naïve quiescent and cycling LSCs are highly sensitive to chemotherapy: Therapy‐naïve quiescent LSCs have low ROS levels and OXPHOS dependent on high BCL‐2 levels when compared to cycling LSCs and bulk AML cells, of which also depend on aerobic glycolysis. Cells which remain postchemotherapy, therapy‐resistant, have notably higher ROS levels, high OXPHOS and FAO when compared to therapy‐naïve AML cells.

AML arises from and is maintained by LSCs [4]. LSCs have been defined by a number of characteristics and in adult AML through the use of xenotransplantation approaches. These include cell surface marker expression (e.g. CD34 and CD38, with many more still being discovered), enhanced/acquired self‐renewal, slow cycling or quiescence, serial engraftment and distinct biochemical features (reviewed in Ref. [77]). Biochemically, AML LSCs defined by cell surface markers or quiescence have low levels of reactive oxygen species (ROS) compared with the bulk blast cycling AML population [78, 79, 80]. Therapy‐naïve quiescent LSCs are characterised by a low rate of energy metabolism with low ROS levels and low mitochondrial oxidative phosphorylation (OXPHOS) dependent on BCL‐2 levels and amino acid metabolism compared to bulk AML blast cells [26, 78]. In contrast, the bulk AML blasts present high glycolytic metabolism affecting cell proliferation and cell survival pathways (Fig. 3) [46, 81]. ROS‐low defined LSCs are unable to upregulate glycolysis and therefore have an increased sensitivity to strategies that block OXPHOS [82, 83]. Such features are being targeted in order to eradicate the AML LSC, an approach thought to achieve improved outcomes for AML. In fact, LSC frequency and associated transcriptional signatures carry clinical prognostic impact and have been extensively associated with adult AML chemoresistance and leukaemia relapse [84, 85, 86, 87].

However, there remains a paucity of studies defining true LSCs specifically in paediatric AML for their eradication. It is common practice to extrapolate from adult studies onto paediatric AML, for example when characterising LSCs using cell surface markers or cycling properties [2, 28] for their biochemical and molecular analysis. Using aberrantly expressed cell surface markers within the CD34^+^/CD38^−/low^ fraction of paediatric AML patients, LSCs were found to be present at a higher frequency at diagnosis in patients who are more likely to fail treatment regimens [88]. A panel of LSC markers in both paediatric and adult AML is necessary given their heterogenous expression, and therefore, sensitivity of LSC frequencies is dependent on the panel used [89]. We are still learning the use of LSC markers for detecting MRD with flow cytometry, and this is being addressed in the paediatric AML Myechild clinical trial (NCT02724163). It is worth considering though that in the absence of definitive xenotransplantation experiments with LSCs defined using cell surface marker expression from paediatric AML, and specifically from the distinct subgroups of AML found in the paediatric population, that important paediatric‐specific features may be masked. For example, one of the most common AML subgroups in the paediatric population, namely MLLr AML, has been distinguished for the presence of cycling, CD93‐positive LSCs within the typically gated CD34^+^CD38^−^ LSC population. However, these findings were reported in a patient cohort that had an average age of 51, with the youngest patient 18 years [90]. We can extrapolate but cannot be definitive that this is true for paediatric MLLr AML. Moreover, adult LSC functionality has also been found in the CD34^−^ fraction [91]; therefore, caution is warranted when assigning and extrapolating LSC characteristics from adult to paediatric AML.

Molecularly, the transcriptional state of the adult AML LSCs distinguishes them from the bulk blast population and nonmalignant HSCs. Microarray transcriptional analysis of adult LSCs was used to establish and validate a 17‐gene stemness score developed using adult outcome data (LSC17) [85]. It is important to note that this score was developed using xenotransplantation of CD34^+/−^CD38^+/−^ sorted fractions. The LSC17 score could predict disease outcome in adult patients with AML in TCGA validation cohorts that contain both microarray and RNA sequencing [60] and could notably also predict disease outcome in the paediatric ELAM02 cohort [92], based on the assumption that paediatric and adult LSCs share common gene expression programmes. The clinical utility of the LSC17 score is being trialled with a Nanostring platform assay to determine prospectively the feasibility and prognostic power of LSC17 score testing in newly diagnosed AML patients [93]. Use of a weight‐adapted LSC17 subscore specific for some subsets of risk groups, as, for example, a low molecular risk groups with a poorer prognosis [94] is anticipated to potentially identify patients with a higher risk of drug resistance at diagnosis that may benefit from novel upfront treatment strategies. This is particularly promising, as predicting therapy resistance to standard therapy in AML patients has been very difficult even with more recent mutational and cytogenetic information [95].

A paediatric 6 gene LSC score (pLSC6) was recently developed [96] using microarray data from 163 paediatric patients that included the most common paediatric subgroups (i.e. core‐binding factor groups, MLLr). The pLSC6 score was selected starting from the same 48 LSC gene pool that were used to generate the adult LSC17 score. Therefore, the pLSC and adult LSC17 scores have four genes in common. The pLSC6 score has been validated with the RNA‐sequencing data from the TARGET database (Box 6), where it showed improved prognostic performance compared to MRD‐ and molecular‐based risk classification, as well as compared to LSC17. Future studies directed towards generation of gene expression profiles in the fraction of paediatric LSCs that have been functionally defined (whether these are exclusively within the CD34^+/−^CD38^+/−^ subset or not) is warranted to further refine the gene expression score specific to paediatric AML, especially as the prognosis of paediatric AML is typically better than that of adult AML.

Box 6 **TARGET**

The Therapeutically Applicable Research to Generate Effective Treatments (TARGET) initiative employed comprehensive molecular characterisation to determine the genetic changes that drive the initiation and progression of hard‐to‐treat childhood cancers. TARGET researchers utilised various sequencing and array‐based methods to examine the genomes, transcriptomes, and for some diseases epigenomes of select childhood cancers. This ‘multi‐omic’ approach generated comprehensive profiles of molecular alterations for each cancer type [31, 32, 97, 98].

### 6.2 LSCs as therapeutic targets

There have been some examples of success in developing anti‐LSC compounds [99, 100, 101, 102, 103]. The approach in general includes using the therapy‐naïve LSC transcriptional profile that distinguishes LSCs from the bulk AML cells and from normal HSCs. Useful data sets for such approaches include those from LSC populations that have been validated by xenotransplantation and tightly linked to poor survival in AML and failure of standard therapy across all AML subtypes [84, 85]. By querying these signatures against a repository of drug candidates, it is possible to identify relevant candidates that could be repositioned for use in AML. Examples include compounds such as antihistamines, cardiac glycosides and glucocorticoids [99]. With regard to chemosensitivity in specific AML cytogenetic and mutational subgroups, there has been recent advances from the BEAT AML cohort [33]. Of particular note, there was a strong association between mutations in *NRAS* and MAPK sensitivity which is interesting as *NRAS* mutations are found commonly in paediatric and adult AML. More adult AML‐associated *IDH2* mutations conferred sensitivity to a broad spectrum of drugs, whereas mutations in *IDH1* conferred resistance to most drugs. Mutations in *RUNX1*, again commonly observed in paediatric and adult AML, correlated with sensitivity to PIK3C and mTOR inhibitors (such as BEZ235) and to the multikinase vascular endothelial growth factor receptor (VEGFR) inhibitor, cediranib.

Another approach in developing novel therapeutic strategies is to use the mRNA expression profile induced upon oncogene expression (e.g. MLL‐AF9, HOXA9 and MEIS1) to screen using Connectivity Maps [104] to identify clinically approved drugs that may revert an early leukaemic transformation gene signature. Histone deacetylase inhibitors were identified in this way [105], which have proved to be good combination therapeutic approaches in adults with AML. Interestingly, antihistamines again were found to have antineoplastic effects via disruption of lysosomal and mitochondrial homeostasis [106]. This link with metabolism is interesting, given the emerging role of metabolic reprogramming in chemotherapy resistance in AML that will be discussed below.

### 6.3 Molecular profiles of chemoresistant cells

Until recently, the LSC subpopulation was viewed as the main source of chemoresistant cells. It has been shown that LSCs are relatively resistant to standard therapies [107, 108, 109] and responsible for driving disease relapse. Therefore, LSCs were pointed out as a clinically relevant therapeutic target for improving AML outcomes. Furthermore, chemoresistant cells were identified as CD34^+^CD38^−^ cells within the osteoblast‐rich endosteal region of the BM [109]. In this paper, global transcriptional analysis of the LSCs that were serially xenotransplanted was used to define features that enabled LSC activity, but not features of chemoresistance. Therefore, it is clear that LSC molecular profiles are stable through xenotransplantation but whether these are the features that result in disease relapse is still a matter of discussion. The question remains whether cells adapt their transcriptional profiles following exposure to chemotherapy or whether there are inherent features in a clone of cells, that may or may not be within the therapy‐naïve LSC compartment responsible for chemoresistance.

**Fig. 4:**
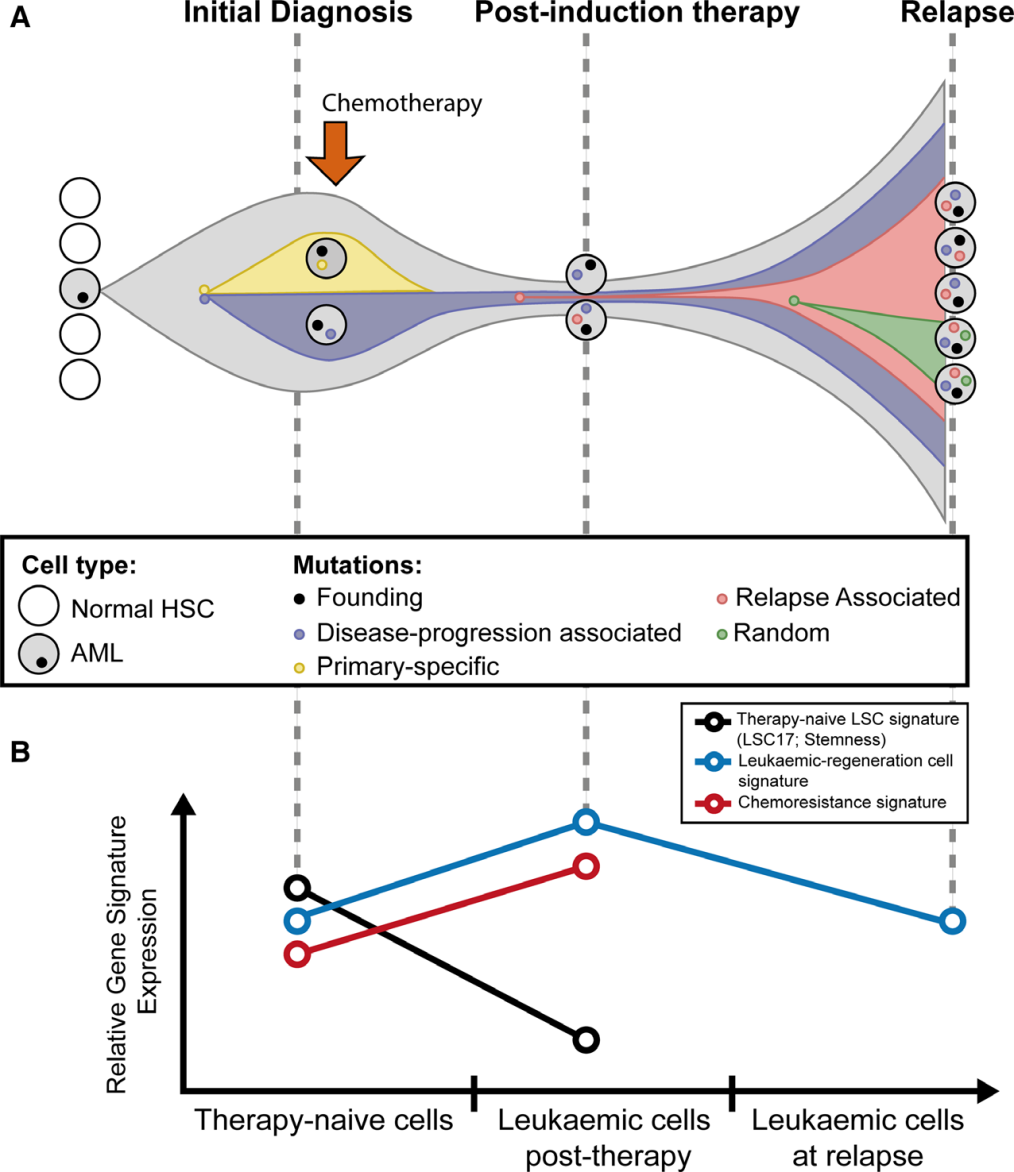
Simple diagrammatical display of *de novo* AML clonal evolution and distinction of gene signatures between therapy‐naïve cells and chemotherapy‐treated cells. (A) The leukaemia‐initiating (founding) clone contains AML pathogenic somatic mutations; among the founding clone, one subclone is eradicated by the chemotherapy whilst the other with relapse‐initiating mutations accumulated further mutations to evolve into a dominant clone at relapse. HSC (adapted from [13]). (B) Molecular signatures, such as therapy‐naïve leukaemia stem cell (LSC) signatures, LSC17 [85] and stemness [84], leukaemic regeneration cell signature [27] and chemoresistance signature [26], are discrete between therapy‐naïve LSCs and leukaemic cells after exposure to chemotherapy during the course of disease in adults. These signatures permit the identification of therapy‐naïve LSCs and the discrimination between impending relapse vs durable disease‐free survival in human AML patients during remission states. The status of these signatures in relapse patients is not yet well defined.

Some light has been shed on the chemoresistant cells using postchemotherapy transcriptional profiling. We now know from adult AML studies that functionally and phenotypically defined LSCs are not enriched with chemotherapy‐resistant cells [26, 27, 110]. Indeed it was shown that LSCs are not resistant to standard chemotherapy [26]. The cells that remain postchemotherapy are distinct from therapy‐naïve LSCs; chemoresistant adult AML cells are strongly driven by cellular metabolism displaying a dependence on fatty acid oxidation (FAO) together with elevated ROS and OXPHOS and a mRNA signature distinct from therapy‐naïve LSCs (Fig. 4B). However, these signatures are temporary, as the cells that are detectable at a clinical relapse stage lack these signatures. It remains to be shown whether such chemoresistant cells re‐acquire the therapy‐naïve LSC transcriptional state at relapse. These molecular features highlight a window of opportunity that exists to target the molecular state of the chemoresistant cell prior to onset of relapse. Targeting mitochondrial oxidative metabolism with OXPHOS inhibitors redirects metabolism towards glycolysis and sensitises resistant cells to standard chemotherapy [26]. Another study showed that inhibition of myeloperoxidase sensitised chemoresistant AML cells to chemotherapy by impairing OXPHOS and cellular energetic balance, triggering oxidative damage and sustaining oxidative stress [111]. The chemoresistant molecular signature links to clinical outcomes in adult AML relapse [26, 27]. Whether this transient transcriptional state is similarly seen in paediatric AML subgroups remains an open question and is most likely different based on the cytogenetic and mutational profiles of the adult AML specimens in those studies, which are not the common paediatric subgroups.

One subgroup of AML patients where biochemical data relating to chemoresistance may be more reliably extrapolated is the *FLT3* mutated subgroup. *FLT3* mutations occur at a similar frequency in ~ 30% of AML patients across the age‐spectrum and is associated with an inferior prognostic outlook [112]. The AML *FLT3‐ITD* mutation has been described to potentiate the Warburg effect, a well‐characterised phenomenon within the field of cancer biology that describes the marked dependency of malignant cells on aerobic glycolysis over OXPHOS [48]. Hence, *FLT3* mutational status represents an attractive focus for the development of future therapies in which subtype‐specific metabolic vulnerabilities can be exploited. Of note, therapeutic intervention with the glycolytic inhibitor 2‐deoxyglucose (2‐DG) has been reported to induce cell death and exert antiproliferative effects in *FLT3‐ITD* AML *in vitro* through a marked inhibition of aerobic glycolysis. Interestingly, 2‐DG‐induced inhibition of glycolysis has also been documented to potentiate the cytotoxicity of conventional Ara‐C chemotherapy as well as restoring chemosensitivity in therapy‐resistant *FLT3‐ITD* AML cells [48, 113]. Despite the promise of these findings, results are primarily based upon observations in cell lines. Thus, inhibiting glycolysis warrants further investigation to define the clinical applicability in large cohorts of both paediatric and adult *FLT3‐ITD* AML patients.

As part of the TARGET initiative, whole‐genome DNA and RNA sequencing was performed on a cohort of paediatric AML patients that failed induction chemotherapy [72]. Distinct and invariant gene expression programmes before and after chemotherapy were seen, as well as diverse forms of clonal evolution upon chemotherapy exposure. Consistent with adult data, there was a relative depletion of clones with *FLT3* mutations. Unsupervised hierarchical clustering of gene expression profiles did not segregate with genetically defined groups. However, Gene Set Enrichment Analysis (GSEA) analysis of primary chemoresistance included programmes positively enriched for OXPHOS, FAO, ROS, G2M checkpoint, MYC targets and E2F targets.

Another contributor to chemoresistance is the BM niche, which changes considerably with age [114], [115]. It has been established that the BM niche supports leukaemic cell metabolism pathways leading to leukaemia chemoresistance [116, 117]. Of note, chemoresistance via interaction between the BM mesenchymal stromal cell and the AML blast cell could be abrogated using an approach that extinguishes energy metabolism in the blast cell [118]. This link with energy metabolism is a common theme with recent chemoresistance insights.

## 7 Conclusions and Perspectives

Challenges remain including assessing the correlations between leukaemia‐specific markers across the different cytogenetic and mutational subgroups in adult and paediatric AML, not only in identifying the surface markers that can aid subclone identification but also MRD detection that could be used to guide patient‐specific treatment strategies, which potentially involve targeting the leukaemia‐specific transcriptional and metabolic programmes. It is clear that paediatric AML is a disease distinguishable from adult AML with regard to the gene mutation spectrum. However, current risk stratification approach only includes common mutations based on prognostic values when the total number of discovered mutations surpasses that. It may be more clinically significant to consider allelic burden profiles of AML‐associated mutations (e.g. targeting genomic abnormalities with high diagnosis VAF) in order to decide on targeted therapies and risk categorisation. It is also important to closely study novel mutations and transcriptional profiles post‐therapy so as to gain perspectives on clonal evolution and instrumentation to relapse. Indeed, targeted sequencing of known mutations is limiting and may not be optimal for advanced disease, therapy‐related and secondary disease or for paediatric subgroups. The ensuing interpretation of the molecular profile in AML patients across the age range and most critically at different phases of disease (i.e. diagnosis, remission and relapse) can be prominent in better appreciating the disease pathogenesis and promise an improvement in patient management approaches. Moreover, conventional combination therapies to target the chimeric clonal subpopulations have a possibility of precarious effects since either pre‐existing or acquired therapy‐resistant clones could proliferate and prevail to result in a fatal relapse. Transcriptional profiling has revealed significant and targetable features of AML cells and being able to identify and specifically kill these chemoresistant clones is the current challenge. Efforts across the globe including Genomics England are endeavourering to bring together ‘omic’ profiling and clinical data to better inform on best treatments for individual patients.

## Conflict of interest

The authors declare no conflict of interest.
